# Evaluating changes in firefighter urinary metabolomes after structural fires: an untargeted, high resolution approach

**DOI:** 10.1038/s41598-023-47799-x

**Published:** 2023-11-27

**Authors:** Melissa A. Furlong, Tuo Liu, Justin M. Snider, Malak M. Tfaily, Christian Itson, Shawn Beitel, Krishna Parsawar, Kristen Keck, James Galligan, Douglas I. Walker, John J. Gulotta, Jefferey L. Burgess

**Affiliations:** 1 Environmental Health Sciences, Mel and Enid Zuckerman College of Public Health, University of Arizona, 1295 N Martin Ave, Tucson, AZ 85724 USA; 2 School of Nutritional Sciences and Wellness, University of Arizona, Tucson, USA; 3 University of Arizona Cancer Center, Tucson, USA; 4 Department of Environmental Science, University of Arizona, Tucson, USA; 5 Analytical and Biological Mass Spectrometry Core, University of Arizona, Tucson, USA; 6 College of Pharmacy, University of Arizona, Tucson, USA; 7 Gangarosa Department of Environmental Health, Emory University Rollins School of Public Health, Atlanta, GA USA; 8 Tucson Fire Department, Tucson, AZ USA

**Keywords:** Metabolomics, Risk factors

## Abstract

Firefighters have elevated rates of urinary tract cancers and other adverse health outcomes, which may be attributable to environmental occupational exposures. Untargeted metabolomics was applied to characterize this suite of environmental exposures and biological changes in response to occupational firefighting. 200 urine samples from 100 firefighters collected at baseline and two to four hours post-fire were analyzed using untargeted liquid-chromatography and high-resolution mass spectrometry. Changes in metabolite abundance after a fire were estimated with fixed effects linear regression, with false discovery rate (FDR) adjustment. Partial least squares discriminant analysis (PLS-DA) was also used, and variable important projection (VIP) scores were extracted. Systemic changes were evaluated using pathway enrichment for highly discriminating metabolites. Metabolome-wide-association-study (MWAS) identified 268 metabolites associated with firefighting activity at FDR q < 0.05. Of these, 20 were annotated with high confidence, including the amino acids taurine, proline, and betaine; the indoles kynurenic acid and indole-3-acetic acid; the known uremic toxins trimethylamine n-oxide and hippuric acid; and the hormone 7a-hydroxytestosterone. Partial least squares discriminant analysis (PLS-DA) additionally implicated choline, cortisol, and other hormones. Significant pathways included metabolism of urea cycle/amino group, alanine and aspartate, aspartate and asparagine, vitamin b3 (nicotinate and nicotinamide), and arginine and proline. Firefighters show a broad metabolic response to fires, including altered excretion of indole compounds and uremic toxins. Implicated pathways and features, particularly uremic toxins, may be important regulators of firefighter’s increased risk for urinary tract cancers.

## Introduction

Firefighting was recently reclassified as a Group 1 Carcinogen by the International Agency for Research in Cancer^1^, and firefighters have elevated rates of multiple cancers and other chronic health conditions, including a 16% increase for bladder cancer^2^, and a 27–30% increase in kidney cancer^3–6^. Environmental exposures encountered during fireground encounters and other occupational activities are presumed to contribute to these risks. These exposures include volatile organic compounds such as benzene, toluene, ethylbenzene and xylene (BTEX)^7–9^, per- and polyfluoroalkyl substances (PFAS), which are found in some Class-B aqueous film-forming firefighting foams; polycyclic aromatic hydrocarbons (PAHs) and PAH-like compounds^10–12^, which are combustion byproducts that increase aryl hydrocarbon receptor (AhR) activity; and organophosphate and organobrominated flame retardants. Firefighters are likely also exposed to a range of other known and unknown environmental compounds while fighting fires.

Although there are documented associations of these specific chemicals with a range of health outcomes^13–15^, the effects of exposure to complex mixture of these chemicals and corresponding biological impacts exposures is unknown. Untargeted metabolomic profiling, which aims to systematically measure thousands of exogenous and endogenous metabolites, can provide key insight into subtle signatures of cancer and other diseases, often before they become clinically apparent^16–18^. In this untargeted metabolomics study, untargeted liquid chromatography was performed with high-resolution mass spectrometry (LC-HRMS) of urine samples collected from 100 Tucson, Arizona male firefighters at baseline and after exposure to structural fires, to evaluate the range of exogenous and endogenous metabolites that change after fire exposures.

## Results

### Study population

The demographics of the 100 participating fire fighters are presented in Table 1. The majority of participants were non-Hispanic white (81%) with a mean age of 38 years.

**Table 1:** Sample characteristics of 100 participants, with samples at baseline and post-fire (200 samples).

Ethnicity	
Hispanic	19 (19.0%)
Not Hispanic	81 (81.0%)
Age (years)	
Median	38
Q1, Q3	30.0, 43.0
Days in storage	
Median, baseline	1815.7
Q1, Q3, baseline	1697.7, 1876.5
Median, post-fire	1849.7
Q1, Q3, post-fire	1751.7, 1888.7

### Metabolome-wide association study

**Figure 1:**
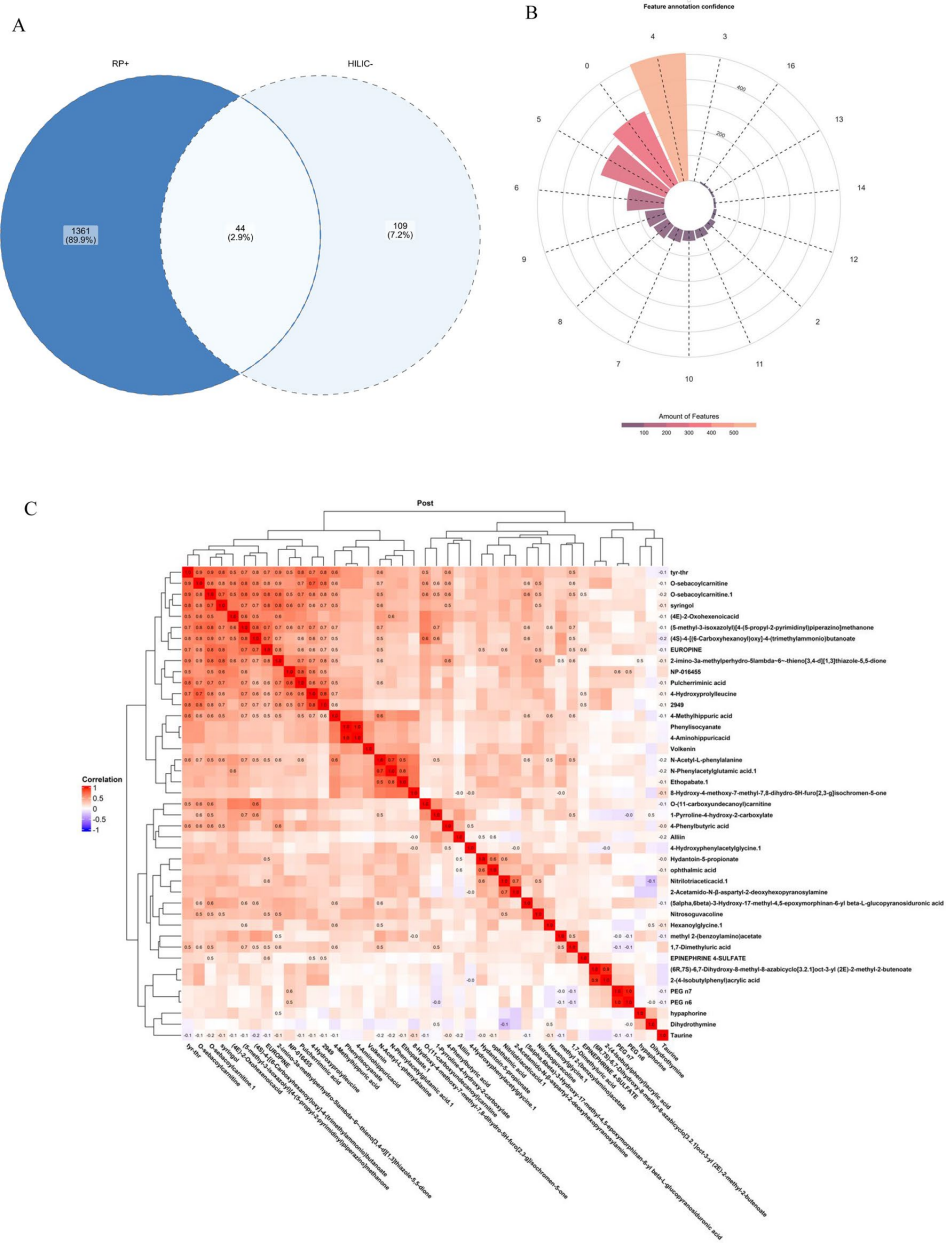
Description of identified metabolomic features. (**A**) Venn diagram of features identified with reverse phase positive mode and HILIC negative mode. (**B**) Distribution of features by confidence level. (**C**) Heat Map and Dendrogram of Correlations among features. Correlation matrix was calculated with Pearson coefficients. Cells in red indicated positive correlations, and blue indicates negative correlations. The strength of correlation was mapped according to color intensity, as shown in legends.

Compound Discoverer denoted 17,192 and 60,139 features in hydrophilic interaction liquid chromatography-negative mode (HILIC−) and reverse phase positive mode (RP+), respectively. 286 metabolomic features from the HILIC− analysis and 3485 metabolomic features from the RP+ analysis passed filters utilizing the multiple QC sample run. Of these features, 175 in HILIC− and 1848 in RP+ produced named annotations. Following filtering for missing values and replicate CV, 1558 metabolomic features (153 HILIC− and 1405 RP+) remained for analyses (Fig. 1a). Among these, 44 (2.9%) were annotated in both HILIC− and RP+ modes. Most features (1220) displayed acceptable confidence (≥ 4, Fig. 1b), although 314 features had low confidence levels of 0.

The heatmap and correlation dendrogram shows the descriptive correlations between the post-fire raw abundances only (Fig. 1c). Clustering patterns are evident amongst this group, with some features displaying minimal correlation patterns; notably, taurine shows few correlations with other metabolites. Syringol, an oxygenated aromatic and component of wood smoke, clusters with pulcherriminic acid, which is formed by oxidative aromatization, and 4-methylhippuric acid, a metabolite of xylene. This cluster may be indicative of smoke-derived metabolites. Most correlations were weak to moderate.

After adjusting for covariates (batch, log specific gravity, and participant) in the fixed effects linear regression models, 268 features were identified that were significantly different post-fire compared to baseline at FDR q < 0.05 (Supplemental Table 1). Of these, 19 unique features, and 20 overall features (taurine was identified in both RP+ and HILIC− modes) had high confidence (≥ 10) (Table 2). These included several amino acids, including taurine, betaine, l-glutamic acid, creatinine, and proline. Hippuric acid (a metabolite of xylene, although it is also a metabolite of other phenolic compounds like fruit juice, tea, and wine) was also observed, and evidence of uremic toxins (*N*-methyl-2-pyridone-5-carboxamide, trimethylamine *n*-oxide (TMAO), and indoles). Among these, 19 features were increased after a fire, and 1 feature (betaine) decreased. In-depth descriptions from the literature of these metabolites are included in Supplemental Table 2.

**Table 2:** High-confidence metabolomic features associated with fireground exposure in fixed-effects linear regression models.

Annotated metabolite	Beta coef	FDR	Conf	Mol. wt	Ret. time	Formula	Mode
Taurine	0.796	0.004	16	125.01464	1.104	C_2_H_7_NO_3_S	RP
Taurine	0.587	0.016	16	125.01469	9.673	C_2_H_7_NO_3_S	HILIC
1,7-Dimethyluric acid	0.517	0.035	11	196.05966	8.881	C_7_H_8_N_4_O_3_	RP
4-Phenylbutyric acid	0.473	0.007	10	164.08372	10.493	C_10_H_12_O_2_	RP
Indole-3-acetic acid	0.435	0.005	10	175.06334	10.801	C_10_H_9_NO_2_	RP
*N*6,*N*6,*N*6-trimethyl-l-lysine	0.410	0.001	10	188.15251	1.076	C_9_H_20_N_2_O_2_	RP
Trimethylamine *N*-oxide	0.357	0.029	11	75.06844	1.162	C_3_H_9_NO	RP
Phenylacetyl-l-glutamine	0.344	0.006	13	264.11097	9.953	C_13_H_16_N_2_O_4_	RP
5-(Acetylamino)-2-hydroxybenzoic acid	0.333	0.001	10	195.05316	9.67	C_9_H_9_NO_4_	RP
Trans-urocanic acid	0.330	0.021	12	138.04291	2.308	C_6_H_6_N_2_O_2_	RP
Hippuric acid	0.330	0.025	10	179.05832	9.792	C_9_H_9_NO_3_	RP
4-Acetamidobutanoic acid	0.290	0.009	10	145.07387	2.418	C_6_H_11_NO_3_	HILIC
l-Glutamic acid	0.277	0.008	11	147.05318	1.164	C_5_H_9_NO_4_	RP
Proline.1	0.266	0.021	11	115.06328	1.33	C_5_H_9_NO_2_	RP
*N*-Acetyl-l-arginine dihydrate	0.254	0.011	12	216.12222	10.826	C_8_H_16_N_4_O_3_	HILIC
Creatinine	0.252	0.012	13	113.05887	1.203	C_4_H_7_N_3_O	RP
*N*6-acetyl-l-lysine	0.239	0.007	11	188.11618	1.392	C_8_H_16_N_2_ O_3_	RP
Kynurenic acid	0.234	0.033	10	189.0426	9.917	C_10_H_7_NO_3_	RP
7a-Hydroxytestosterone	0.196	0.01	10	304.20386	15.775	C_19_H_28_O_3_	RP
Betaine	-0.290	0.001	14	117.07892	1.187	C_5_H_11_NO_2_	RP

Results are from fixed effects linear regressions estimating the effect of sample type (post-fire vs pre-fire) on log2 metabolite abundance. Models were adjusted for batch, log specific gravity, and participant number. Only features with confidence > 10 are shown here, which corresponds to MSI identification levels of 1 or 2^74^. A beta coefficient of 0.45 corresponds to a fold change of 1.36, and a beta coefficient of −0.45 corresponds to a fold change of 0.732. The full list of features that met FDR < 0.05 threshold are included in the Appendix.

Several features were identified that potentially reflect environmental sources (Supplementary Table 1). These include a range of naphthols, syringol (a component of wood smoke), europine (a hepatotoxic pyrrolizidine alkaloid), a benzene diamine, and metabolites of xylene (4-methylhippuric acid and 3-methyl hippuric acid).

**Figure 2:**
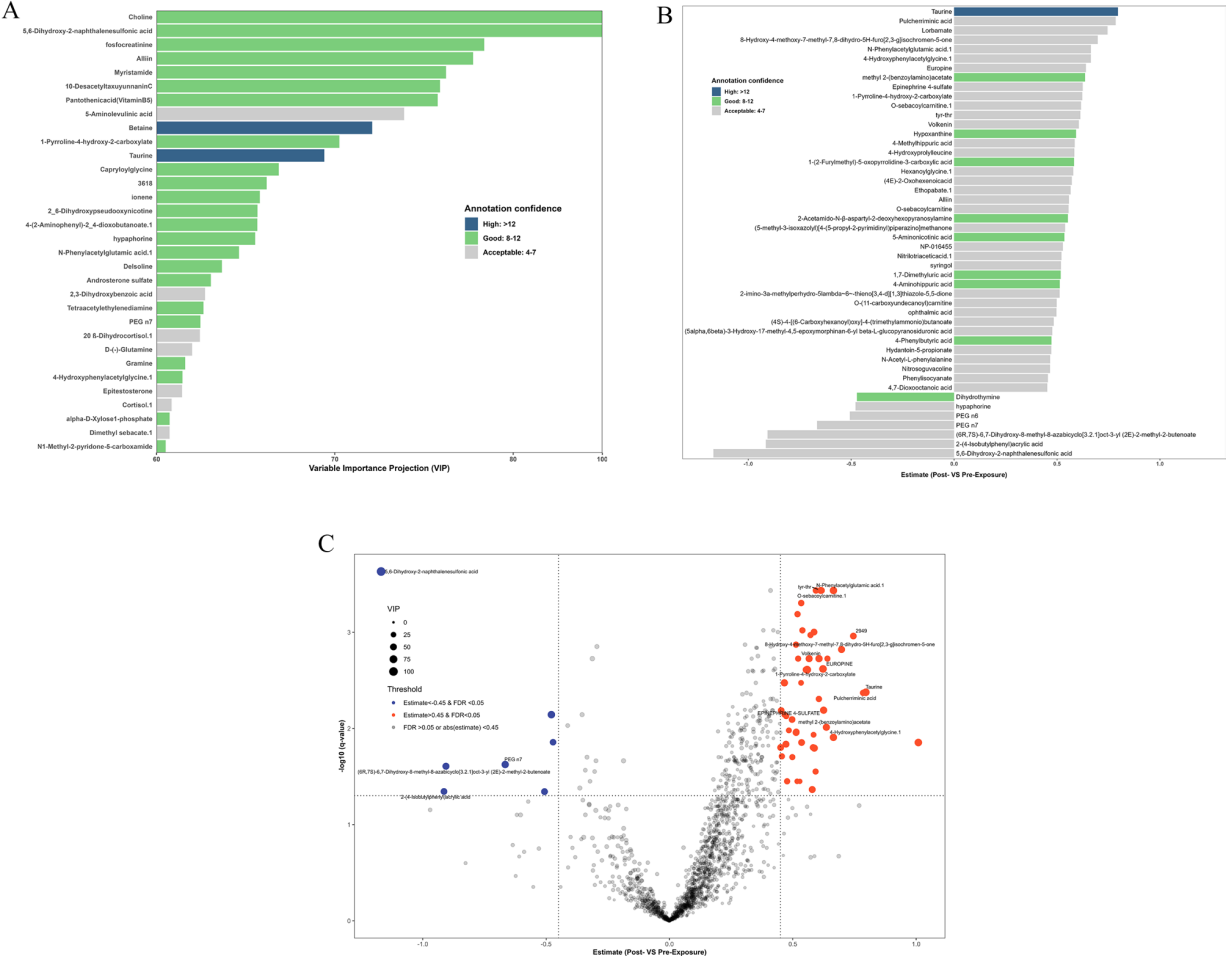
Metabolomic features with high fold-changes and annotation confidence from pre-fire to post fire. (**A**) Features identified with high variable importance projection (VIP) values(≥ 60) in the PLS-DA model with acceptable confidence (> 3), discriminating between pre and post-fire values. (**B**) Waterfall plot of features identified from the linear regressions, showing differences for post-fire versus pre-fire (x axis linear regression coefficient estimates of post-fire to pre-fire, for those with high fold changes (estimate > 0.45 or < −0.45, confidence > 3, and FDR q value < 0.05). (**C**) Volcano plot of metabolomic feature levels of post-fire versus pre-fire (x axis log2 fold change of post-fire to pre-fire; y axis, log10 FDR adjusted p-value). Metabolites with VIP scores on PLSDA ≥ 20, fold change ≥ 1.5, and FDR q-value < 0.05 are plotted in red and those with VIPplsda ≥ 20, fold change ≤ 0.8, FDR q-value < 0.05 in blue.

The partial least squares discriminant analysis (PLS-DA) model displayed good classification performance with a median accuracy of 0.725 (0.70 for HILIC- and 0.75 for RP+). The classification model-based feature selection approach identified 38 features with VIP > 60 and confidence > 3 (7 from HILIC- and 31 from RP+, Fig. 2C). Individual features from these models, and from linear regression models (FDR < 0.05, estimates > 0.45 or < −0.45, and confidence > 3) are presented in Fig. 2a–c. The PLS-DA model (Fig. 2a) identified two features with very high annotation confidence (betaine and taurine), and several features with good confidence. Of these, the three with the highest variable importance in projection (VIP) scores included choline, 5,6-dihydroxy-2-naphthalenesulfonic acid, and phosphorylated creatinine (labelled as fosfocreatinine). For linear regression (Fig. 2b), taurine had the strongest positive estimate and the highest confidence. Other features with high estimates included pulcherriminic acid, 2949, *n*-phenylacetylglutamic acid, europine, and 5,6-dihydroxy-2-naphthalene sulfonic acid. We observed overlap among eight features identified with acceptable confidence from the linear regression and PLS-DA models. These included taurine, N-phenylacetylglutamic acid, 4-hydroxyphenylacetylglycine, 5,6-dihydroxy-2-naphthalenesulfonic acid, 1-pyrroline-4-hydroxy-2-carboxylate, aliin, hypaphorine, and PEG N7. In the volcano plot (Fig. 2c), features that clustered with acceptable confidence, high estimate changes and low p-values included o-sebacoylcarnitine, tyr-thr (l-tyrosyl-l-threonine), and n-phenylacetylglutamic acid.

**Figure 3:**
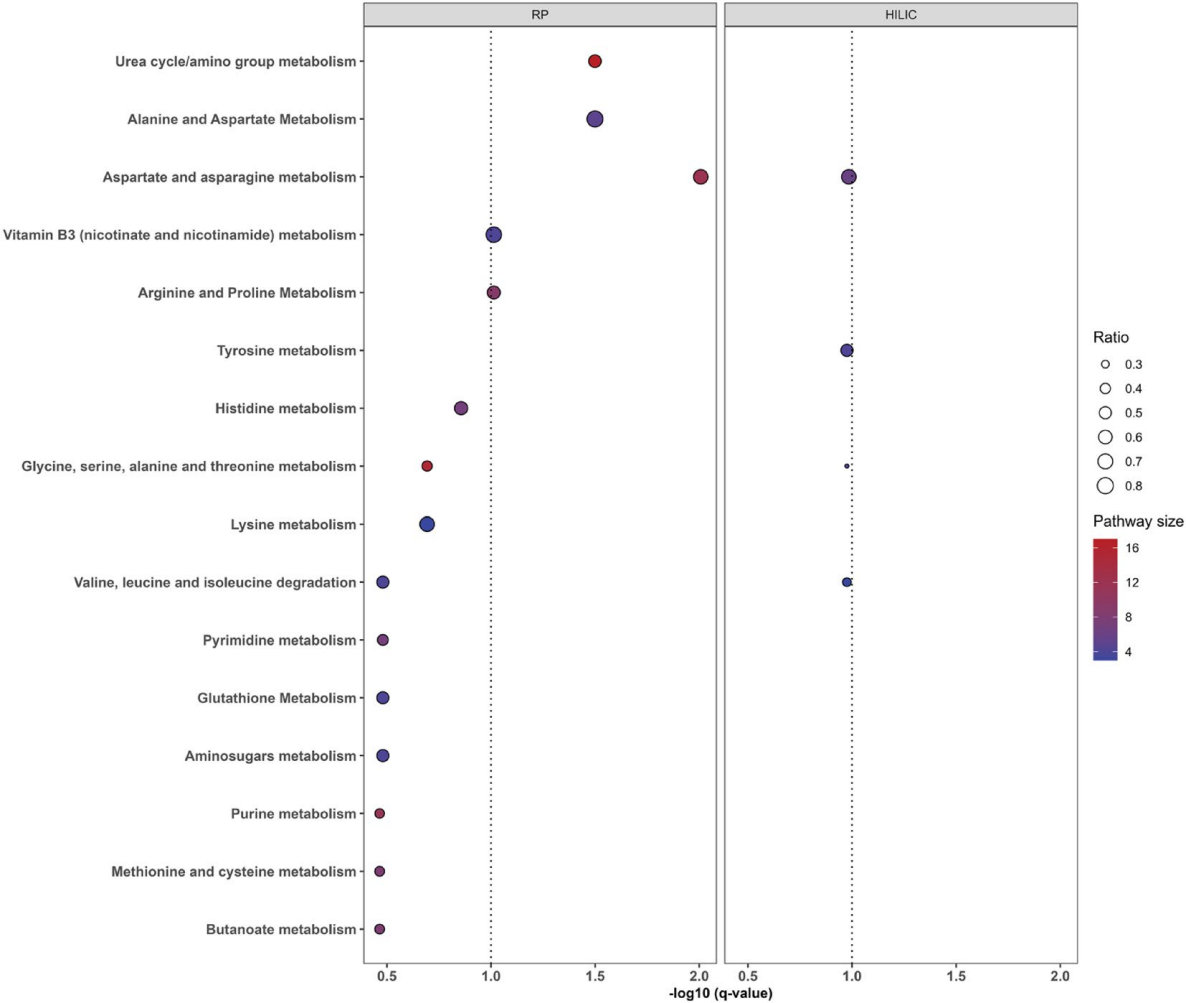
Pathway overrepresentation analysis of post-exposure versus baseline based on features with VIP > 20 in PLS-DA models; dashed line indicates FDR q-value = 0.1; ratio represents the ratio of the number of significant metabolites to the number of total metabolites present within the pathway (pathway size); only pathways with pathway size ≥ 3 and at least one overlapping metabolite are presented. Mummichog reannotates features based on their own internal library, so feature IDs may not exactly overlap with those reported in feature-specific tables.

Mummichog^19^ was used to test whether features that were selected by PLS-DA classification were enriched for specific metabolic pathways (Fig. 3). Five pathways were significantly enriched after FDR adjustment at level of 0.1, including aspartate and asparagine metabolism, urea cycle/amino group metabolism, alanine and aspartate metabolism, vitamin B3 (nicotinate and nicotinamide) metabolism, and arginine and proline metabolism. No significant enriched pathway was identified from HILIC− mode.

## Discussion

Changes in the urinary metabolome from firefighters from baseline to after a fire are indicative of broad biological responses across the metabolome: changes in several amino acids and amino acid pathways indicative of one-carbon metabolism are reported, as well as changes in hormones, volatile organic compounds (xylene metabolites), indole metabolites related to AhR activity (Indole-3-acetic acid, gramine), and uremic toxins. Elevation of syringol, a primary component found in woodsmoke, was also observed. This untargeted metabolomic approach is the first application of this method to firefighters that identifies changes after a fire, and revealed broad environmental contributions and biological responses after this acute occupational activity.

### Amino acid metabolism and cancer

Firefighting is considered a Group 1 carcinogen by the International Agency for Research on Cancer (IARC)^2^, with strong causal evidence for mesothelioma and bladder cancer, and evidence for other cancers including bladder, kidney, colon, prostate, and testicular cancers, and melanoma and non-Hodgkin lymphoma^2^. In support of this, in this study, several amino acids and pathways were indicative of one carbon metabolism. Cancer cells characteristically rely on aerobic glycolysis (i.e. the Warburg Effect), producing lactate at the expense of oxidative metabolism (i.e. TCA cycle). As the TCA cycle provides critical metabolic intermediates necessary for cell health, many cancers are capable of shifting metabolism to support this pathway^20^. This is largely achieved through the catabolism of Asp, Gln, Glu, Arg, and Pro^20^. Our data evaluating the urine metabolome of pre- vs. post-exposure demonstrates a significant enrichment in these pathways, potentially drawing a link between fireground exposure*s* and cancer metabolism. In further support of this pro-metastatic metabolic reprogramming, a significant enrichment in pathways associated with purine/pyrimidine metabolism was observed. This is a hallmark of many cancers, where the rapid proliferation rates require increased de novo nucleotide biosynthesis^21^. The critical carbon and nitrogen units required for this process can be derived through catabolism of amino acids, notably Gly, Gln, and Asp, which are significantly elevated in the post-fire cohort^20^.

Interestingly, elevated levels of several known uremic toxins were also observed, which is consistent with a prior study in Saudi Arabian firefighters showing changes in urea nitrogen and kidney functioning after a fire^22^. These include *N*-methyl-2-pyridone-5-carboxamide, trimethylamine n-oxide (TMAO) ^23^, hippuric acid, and the indoles indole-3-acetic acid, tryptophan and tryptophan derivatives (kynurenic acid, hypaphorine)^24^ Disruption of trimethylamine has been strongly implicated in kidney related disease outcomes^25^, and is further generated from choline, betaine, and carnitines, all of which were observed in this study. Tryptophan metabolism enzymes primarily are sourced from the liver, kidney, and brain^26^. Indoles are also related to tryptophan and are tryptophan derivatives, which may reflect general disruption of the tryptophan pathway, and indole disruption may also represent functional changes in the gut^27,28^. This gut based tryptophan metabolism mediates renal fibrosis^29^, which precedes kidney cancer. Some overlap was also observed with features identified in previous untargeted metabolome-wide association studies of bladder cancer: notably taurine^30^, but also choline, aminobutyric acid, dihydrotestosterone, niacinamide, and adenosine^31^. Taurine is related to oxidative stress^32^. Large changes in 4-phenylbutyric acid were also reported. Phenylbutyrate is a derivative of butyric acid, which is produced by colonic bacteria fermentation, and is also a drug currently used to treat urea cycle disorders^33^. This metabolite is conjugated to glutamine and forms phenylacetyglutamine, another high-confidence feature with large changes after fires. These two metabolites may reflect temporal changes in medication usage, or activation of urea cycle pathways. In addition to taurine, changes in hippuric acid, phenylacetylglutamine, and carnitines, were also observed, which have been previously shown to discriminate strongly between bladder and kidney cancer cases and controls^34^. Carnitines are associated with mitochondrial beta-oxidation dysfunction^35^.

It is unknown if these metabolites are on the causal pathway between environmental exposures and cancers and other health outcomes, or if they reflect an altered underlying biological process that occurs during kidney and bladder cancers. For instance, due to the cross-sectional nature of previous untargeted metabolomics studies, it is impossible to know if changes in these metabolites contribute to the disease process or is a metabolic adaptation. Our study suggests that in an occupational group with higher risks of these cancers, these metabolites may be acutely altered after an occupational event in otherwise healthy individuals. If these metabolites are causally related to cancer, then acute, repeated increases in these metabolites over time may be responsible for increased risk of bladder and kidney cancer among firefighters. Alternately, acute, repeated increases in a biological process that results in changes in metabolite excretion may be related to the increased risk. These metabolites may provide a potential point of intervention for reducing cancer risk long-term in firefighters. If so, then intervention in these metabolic pathways with supplements, or long-term monitoring of levels to identify potential elevated risk, might reduce the incidence of urinary tract cancer in this high-risk population.

### Hormones

Somewhat unexpectedly, changes in several sex steroid hormones were observed, including 7a-hydroxytestosterone, epitestosterone glucuronide, estriol, and 5a-androstan-3,6,17-trione, which may be a stress or immune response. Previous studies have reported changes in adrenaline (epinephrine) and norepinephrine ^36^ after a fire, and although both norepinephrine and epinephrine did increase after a fire in our study, they did not meet the FDR < 0.05 cutoff (the FDR q values were 0.07 and 0.08, respectively). Some of these hormonal responses may be due to endocrine-disrupting properties of some environmental chemicals^37–39^. Previous studies have shown that extracts from used firefighter gear displayed strong antiestrogenic effects^40^, and AhR activation, which we have previously shown to occur in firefighters^10^, is associated with estrogenic responses in human cells^41^.

### Environmental exposures

Several findings were consistent with exposures to fires and air pollution. Syringol, for instance, is an important component of wood and charcoal smoke, and was annotated with acceptable confidence (≥ 4). The arginine and proline pathways were upregulated, as well as the individual features *N*-acetyl-l-arginine dihydrate and arginine, and arginine plays a role in response to burns, smoke inhalation, and nitric oxide ^42–47^. Hypoxanthine may be indicative of physical activity^48,49^, as well as aspartate and asparagine metabolism pathways^50,51^. Both arginine and hypoxanthine have also been identified as key features identified in ultra-high resolution metabolome-wide association studies (*MWAS*) of air pollution^52,53^, which is relevant since fire smoke contains multiple components of air pollution at high concentrations. These features and pathways may thus reflect a response to typical fireground exposures and experiences. In targeted analyses of metabolite changes after fires, others have reported increases in the volatile organic compounds (VOCs) xylene, styrene, and benzene^9^. Elevations in methylhippuric acid were also observed, which are metabolites of xylene, and in fact, several features annotated in our data as a form of hippuric acid had FDR q values < 0.10 (4-methylhippuric acid, 4-aminohippuric acid, hippuric acid). Other features identified with potential environmental sources include several naphthalenes, including 1,3,6,8-naphthalenetetrol, a benzenediamine, and a known carcinogen, 1-nitrosopiperidine^54–56^. Naphthalenes and other PAHs have previously shown to be elevated after fires in targeted analyses^12^, and are associated with cancer and other adverse health effects^57^. Although others have reported increases in the specific PAHs 1-hydroxynaphthalene and 1-hydroxyacenapthene^58^, these studies were targeted for PAHs with specialized extractions and analytical techniques. As ours was an untargeted analysis, more generic extractions and less specific chromatography was utilized, and likely impacted our ability to accurately annotate these molecules. Most of the annotated naphthalene features in this study increased after fires, relative to before, with the exception of 5,6-dihydroxy-2-naphthalenesulfonic acid, a feature with a very low p-value, large absolute fold change, and very high VIP value, which actually decreased after a fire. 2-Naphthalene 2-sulfonic acid is used in the synthesis of dyes, food coloring, surfactants, and dispersants. 5,6-dihydroxy-2-naphthalenesulfonic acid may result from hydroxylation of this compound via the CYP450 phase I pathway. If other unannotated compounds which firefighters are highly exposed to, are preferentially hydroxylated first, the decrease in the hydroxylated form of 2-naphthalene sulfonic acid may reflect decreased capacity for phase 1 detoxification, at the time point measured here. Future studies should incorporate multiple time points.

PAHs are AhR ligands, and AhR activity is increased after fires^12^, which we have previously hypothesized to be due to PAHs. However, in a recent bioassay, we found that most AhR activity was not, in fact, due to the hydroxylated PAHs tested^12^, and was instead likely due to other unidentified compounds. Interestingly, indoles display potent AhR activity, and in this study, several indoles were significantly associated with fires, including indole 3-acetic acid and kynurenic acid (a tryptophan metabolite), both of which were described in Table 2. PAHs and other environmental contaminants have also been shown to disrupt tryptophan metabolism^59,60^, and a complex relationship exists between the indoles, PAHs, and AhR^23^. Increased tryptophan and indole activity in response to environmental exposures may be responsible for the enhanced AhR activity after fires. This is further supported by a review of MWAS studies of environmental exposures^60^, where authors identified features most commonly associated with various environmental exposures. Among those features, we observed in our study increases post-fire of various forms and derivatives of tryptophan, but also phenylalanine, proline, methionine, hypoxanthine, tyrosine, arginine, and derivatives of lysine. In a study of PAH exposures in earthworms, lysine was similarly upregulated^61^, and in another study of PAHs in zebrafish, tryptophan pathways were also activated^62^.

### Dementia

Interestingly, tryptophan, kynurenic acid, urea cycling, and kidney functioning have also all been implicated as risk factors for Alzheimer’s disease and other dementias^63–67^. In a metabolomic analysis of features comparing Alzheimer’s disease to controls, phenylacetylglutamine, l-Arginine, hypoxanthine, uric acid/uric acid derivatives, betaine, and cortisol, along with the arginine and proline metabolism pathway, were significantly different in the AD group^68^, and were elevated in our participants after fires.

### Future directions

Overall, changes in features that reflect environmental sources were observed, as well as features that reflect endogenous biological activity. Some of the environmentally derived metabolites may be driving changes in some of these endogenous metabolites. Future steps will involve a deeper dive into these metabolites to identify whether and which environmental contaminants are driving changes in the endogenous metabolites. The two to four hour post-fire collection of urine in this study was selected to maximize concentrations of urinary PAH metabolites, specifically urinary naphthols, which are present at relatively high concentrations^12^. Given that other contaminants in the fire may have longer elimination half-lives, future studies should also examine urine collected at additional time points after the fire.

Taurine was consistently identified with strong fold changes and discriminatory capacity in both PLS-DA and linear regression models. As the dietary intake of the firefighters was not restricted, taurine and some of the other features could be due to ingestion of energy drinks or medications in the immediate post-fire period, although based on discussion with our firefighter coauthors we believe that energy drink consumption is likely limited to approximately 10% of firefighters.

### Strengths and limitations

This study had several strengths and limitations. A within-person, pre/post exposure paradigm was used to potentially identify candidates for acute changes in firefighters that inherently controlled for time-invariant confounders, and two methods of statistical analysis (linear regression and PLS-DA) were applied. Two modes (RP and HILIC) were used to maximize the number of identified features, along with a high resolution Orbitrap Exploris™ 480 Mass Spectrometer. Limitations include validation against a limited in-house library for annotation of features, evaluation of a narrow range of time post-fire, and inability to control for all time-variant confounding, including potentially medications and dietary changes. In future studies, we will evaluate metabolomic changes by Hispanic ethnicity, and the effect of timing of collection post-fire on feature abundance. Although several signals consistent with increased risk for kidney cancer were observed, the sample is young enough and small enough that we are unable to actually evaluate if any of our participants developed cancer. However, identifying increased risks for cancers among firefighters is an important future goal.

## Conclusions

A broad suite of responses to fires in firefighters were identified that implicate urinary tract cancers, one carbon metabolism, xylene and PAH exposures, as well as signals that point towards a complex interplay between PAHs, AhR, indoles, and kidney and bladder cancer.

## Methods

### Study population & sample collection

Firefighters were enrolled as part of a cancer prevention study partnership between the University of Arizona and the Tucson Fire Department (TFD)^12^. The study protocol was approved by the University of Arizona IRB, and all methods were carried out in accordance with relevant guidelines and regulations. Informed consent was obtained from all subjects.

For this metabolomics study, a subset of samples were selected that included 100 male firefighters who donated urine at a time when they had not responded to a fire for at least 4 days (baseline pre- or remote post-fire samples, referred to as baseline hereafter), and who additionally donated urine samples within 2–4 h after responding to a structural fire (post-fire sample). All fires were structural fires in Tucson, and were predominantly residential with some commercial fires. Structure contents were similar across fires, and did not include hazardous materials.

Urine samples were transported from the Tucson Firefighter Department on ice, specific gravities were measured, and urine samples were aliquoted in 1.0 ml aliquots and stored at −80 °C for long-term storage at the University of Arizona. Demographic and occupational questionnaires were administered to participants at study enrollment, which took place between years 2015 and 2016.

### Sample preparation

The urine samples were prepared for analysis by spiking 20 µl of 20 µM ^13^C labeled internal standard mix (containing labeled phenylalanine, succinic acid, valine, taurocholic acid) into a 1:1 solution of urine and ice cold acidified methanol to provide an acidified solution at 0.1% formic acid. The resulting mixtures were vortexed, centrifuged, and the supernatants collected. Samples were extracted in duplicate and stored at −20 °C until high performance liquid chromotagraphy mass spectrometry (HPLC–MS) analysis.

### High resolution metabolomics

Untargeted metabolomic profiling was performed using established methods (previously described in^69^) on a Thermo Scientific Orbitrap Exploris™ 480 high-resolution mass spectrometer interfaced to a Vanquish Duo Ultra High Performance Liquid Chromatography system (Thermo Scientific, Waltham, MA). Samples were extracted and analyzed in duplicate (biological replicate), extracts were injected using a dual-column setup (reverse phase (RP)/hydrophilic interaction liquid chromatography (HILIC)) that included C18 chromatography (reverse phase) with positive electrospray ionization (ESI) (RP+) and hydrophilic interaction chromatography with negative ESI (HILIC−). Analyses were performed in two batches within 3 months of each other. Samples were randomized across batches, with the exception of samples from Hispanic firefighters (n = 21), which were all analyzed during the first batch. We ran three quality controls (QCs) after every 30 injections (15 samples). These three QCs included (1) an internal laboratory sample (ILS-QC) comprised of a mixture of > 50 randomly chosen urine samples, which were extracted with every batch to assess inter-batch variability, (2) a pooled QC that was comprised of a mixture of all extracted samples, and (3) a standards library mix (to access retention time drift). Tandem mass spectrometry (MSMS) data was acquired using iterative MSMS available through AcquireX, which was collected using process blank samples and the pooled QC sample (referenced as #2 above) at the beginning of each batch.

Following a 1 μL sample injection, RP separation was accomplished using a 1.8 µm, 2.1 × 150 mm HSS T3 Column (Acquity Premier HSS T3 Column) and methanol gradient (A = 99.9% water 0.1% formic acid, B = 99.9% water 0.1% formic acid) consisting of an initial 3 min period of 99% A, and 01% B, followed by linear increase to 50% B at 11 min and then increase to 95% B hold for 2 min.

HILIC analyte separation was accomplished using a 1.7 µm, 2.1 mm × 150 mm Amide column (Waters ACQUITY Premier BEH Amide Column) with 10 mM ammonium formate and acetonitrile gradient (A = 10% water, 90% ACN, 10 mM ammonium formate and 0.1% formic acid, B = 50% water, 50% ACN, 10 mM ammonium formate and 0.1% formic acid) consisting of an initial 3 min period of 99% A, and 01% B, followed by a linear increase to 50% B at 11 min and then increased to 95% B hold for 2 min.

Mobile phase flow rate was 0.3 mL/min for both the RP and HILIC methods. The mass spectrometer was operated using ESI mode at a resolving power of 60,000 and mass-to-charge ratio (m/z) range 65–1000 Da. Detection of *m/z* features was accomplished by a maximum injection time of 100 ms and custom AGC target [normalized AGC target (%)] of 50%.

### Annotation

Compound Discoverer 3.2 (Thermo Scientific, Waltham, MA) was used for spectral alignment, and peak picking, identification and annotation, with fill gaps imputation using random forest algorithm. Features were annotated utilizing multiple online databases and an in-house library, but priority annotations were applied from the in-house standards library developed on the exact same analytical platform used for sample analysis. Confirmed metabolites were identified using an in-house library stored in MzVault that was generated using selected authentic standards from MetaSci, Inc’s complete human metabolome library, and includes *m/z*, retention time and MSMS spectra for 840 and 442 metabolites in RP( +) and HILIC(−), respectively. Metabolite annotations was performed using MzCloud, ChemSpider, and Metabolika. After annotating metabolites, manual QC was performed on the features to eliminate poorly annotated features. QC correction was applied in Compound Discoverer using a linear regression model which only retained features with a QC area RSD < 40% and was limited to a maximum correction of < 20%. The internal laboratory samples that were extracted with every batch had < 20% variation in TIC over the run, indicating all batch extractions were comparable. Mass spectra and chromatography from duplicate annotations (for a single compound) were manually inspected for spectral quality, peak shape, and retention time following data processing with Compound Discoverer to access if one of the multiple annotations was correct. When a single confident annotation was identified, all other duplicate feature annotations were removed. After statistical analysis, features meeting the FDR threshold were manually evaluated for chromatographic and spectral quality.

The percentage of missing features and coefficients of variation among biological replicates were calculated (samples that used “gap fill” function and had gap filled, were considered missing), and only included mass spectral features detected in > 25% of all urinary samples, and those with median coefficients of variation between sample replicates < 20%. Duplicate feature intensities were averaged. Exploratory data analysis was conducted to confirm normality of feature intensity. All features were mean centered and scaled by standard variation such that a one unit increase was equivalent to a 1 SD increase, and features with zero variance were removed before being used analyses. To obtain a holistic view of the interactions among metabolites, a correlation matrix was constructed using Pearson correlation coefficients of the raw abundances and visualized the interaction web using Complex Heatmap. To reduce the number of displayed features, the dendrogram was limited to those features selected as important in the analysis of changes from baseline to post-fire in the linear regression models (described below). To augment confidence scores provided by Compound Discoverer, which do not take into account the databases utilized for the annotation (and the accuracy inherent to those searches), a modified confidence score was adopted of annotation based on annotation sources and match strength from our in-house database (MzVault), and online databases (MzCloud, Chemspider, Metabolika, Masslist), described in Supplement 1. Compounds were only ranked based on this scoring mechanism; no compounds were removed from the data set based on this scoring.

### Metabolome-wide association study

To evaluate changes from baseline to post-fire sample collection, a series of fixed effects linear regressions were first performed on the log2 value of abundance for each feature, with a fixed effect for participant. Since fixed effects linear regression inherently accounts for time-invariant confounding, batch and log specific gravity were also adjusted for. False discovery rate (FDR)^70^ correction was applied to the p-values, and associations with FDR q-values < 0.05 are reported. For parsimonious presentation, significant features are presented, with additional filters. First, those features are presented that met FDR significance at q < 0.05, and with very high confidence (≥ 10). Then, all significant features with high coefficient estimates (> 0.45 or < −0.45) are reported, with acceptable confidence (≥ 4) and FDR < 0.05, and in the supplement, all features with FDR < 0.05 are reported, regardless of fold change or confidence.

Since linear regression does not account for potential collinearity between metabolites, a model-based variable selection was additionally performed (Partial Least Squares Discriminant Analysis, or PLS-DA) with R package caret (caret package version 6.0–86). The PLS-DA algorithm is a widely used discriminant analysis tool, due to its versatility and capability in handling high-dimensional data and collinearity^71^. Cross-validation based model accuracy was calculated to assess the validity of the classification model since we have a balanced dataset^72^ (Table 1), and variable importance projection (VIP) was used to quantify feature contribution to the PLS projection^73^. VIP measures the contribution of the coefficients, which are weighted proportionally to the reduction in the sums of squares and is scaled to 0–100 as a default. A fivefold cross-validation was used to better capture classification performance. Since PLS-DA does not inherently adjust for covariates, we first calculated residuals from the fixed effects linear regression model after controlling for participant number and batch. These residuals were then used as input for the PLS-DA classification model. Features with VIP ≥ 60 are presented. VIP scores in the *caret* package are scaled to a maximum score of 100.

All analyses were done using R (version 4.1.2) with all packages freely implemented.

### Pathway analysis

Pathway overrepresentation analysis using Mummichog was conducted (version 2) to identify enriched metabolic pathways associated with fireground exposure. Since overrepresentation analysis requires a relatively large number of features to identify pathways, the cutoff for importance was relaxed in the pathway models to 20 to allow the algorithm sufficient information. Thus, features previously selected by PLS-DA classification with VIP ≥ 20 were included in the pathway overrepresentation analysis. Mummichog predicts metabolite annotation and biological activity directly from mass spectra without upfront metabolite identification (Li et al.^19^). A pathway was considered significant if the adjusted p-values were smaller than 0.1. Only pathways with at least three discriminative metabolites (pathway size/entries ≥ 3) were interpreted. Pathway analysis was also performed using results from the linear regressions and included all features with raw p-values < 0.05.

### Supplementary Information

Supplementary Tables.

## Data Availability

Deidentified data is available from the authors upon request, please contact mfurlong@arizona.edu.
