# Supplementary material for: Evaluating changes in firefighter urinary metabolomes after structural fires: an untargeted, high resolution approach
Source: Sci Rep. 2023 Nov 27;13:20872. doi: 10.1038/s41598-023-47799-x (PMC10682406; doi:10.1038/s41598-023-47799-x)
Supplement: Supplementary file 1 — Supplementary Tables. [file 41598_2023_47799_MOESM1_ESM.pdf]

## Supplementary description 1 Confidence Scoring Mechanism

We implemented the following scoring mechanism: if a full match with all above sources was achieved, a score of 5 for MzCloud, 4 for Chemspider, 3 for Metabolika, and 3 for Masslist, was assigned to the feature; if partial match, a score of 2 was assigned; if no match, then a score of 0 was assigned; a score of 2 was assigned if the mass difference was less than 5 ppm and a score of 0 was assigned if the mass difference was greater than 5 ppm. Consequently, each feature had 5 scores and the sum of them was interpreted as the overall annotation confidence in this case. We categorized scores into the following categories: ≤3 was considered low confidence, ≥4 – 9 are acceptable, and ≥10 was considered high confidence.

*Supplementary Table 1 List Of All Features Identified in Linear Regression with FDR q<0.05*

| Metabolite | Est | FDR | Conf. | Mol Wt | Ret. Time | Formula | Mode |
|---|---|---|---|---|---|---|---|
| Acesulfame potassium | 1.009 | 0.013 | 0 | 162.99391 | 1.435 | C4 H4 N O4 S | H |
| Taurine | 0.796 | 0.004 | 16 | 125.01464 | 1.104 | C2 H7 N O3 S | RP |
| Pulcherriminic acid | 0.784 | 0.004 | 4 | 256.1423 | 12.053 | C12 H20 N2 O4 | RP |
| 2949 | 0.745 | 0.001 | 4 | 258.15795 | 12.918 | C12 H22 N2 O4 | RP |
| 8-Hydroxy-4-methoxy-7-methyl-7,8-dihydro-5H-furo[2,3-g]isochromen-5-one | 0.697 | 0.001 | 4 | 248.06846 | 11.188 | C13 H12 O5 | RP |
| N-Phenylacetylglutamic acid.1 | 0.664 | <0.001 | 4 | 265.09502 | 10.997 | C13 H15 N O5 | RP |
| 4-Hydroxyphenylacetylglycine.1 | 0.664 | 0.012 | 5 | 209.0688 | 10.764 | C10 H11 N O4 | RP |
| Europine | 0.64 | 0.001 | 4 | 329.18397 | 9.883 | C16 H27 N O6 | RP |
| methyl 2-(benzoylamino)acetate | 0.636 | 0.009 | 9 | 193.07389 | 11.22 | C10 H11 N O3 | RP |
| Epinephrine 4-sulfate | 0.625 | 0.006 | 4 | 263.04644 | 9.869 | C9 H13 N O6 S | RP |
| 1-Pyrroline-4-hydroxy-2-carboxylate | 0.622 | 0.002 | 6 | 129.04256 | 6.746 | C5 H7 N O3 | RP |
| O-sebacoylcarnitine.1 | 0.616 | <0.001 | 4 | 345.2152 | 11.935 | C17 H31 N O6 | RP |
| tyr-thr | 0.613 | <0.001 | 4 | 282.12157 | 11.671 | C13 H18 N2 O5 | RP |
| Volkenin | 0.606 | 0.001 | 4 | 287.10052 | 9.219 | C12 H17 N O7 | RP |
| 4'-ethyl-N-(1-ethynylcyclohexyl)[1,1'-biphenyl]-4-carboxamide | 0.605 | 0.004 | 0 | 331.19956 | 10.788 | C23 H25 N O | RP |
| 5,6,7-Trimethoxy-2H-chromen-2-one.1 | 0.593 | <0.001 | 0 | 236.06605 | 11.916 | C12 H12 O5 | RP |
| Hypoxanthine | 0.592 | 0.028 | 9 | 136.03855 | 3.893 | C5 H4 N4 O | H |
| Taurine | 0.587 | 0.016 | 16 | 125.01469 | 9.673 | C2 H7 N O3 S | H |
| 4-Methylhippuric acid | 0.586 | <0.001 | 7 | 193.07389 | 11.888 | C10 H11 N O3 | RP |
| 4-Hydroxyprolylleucine | 0.584 | 0.011 | 4 | 244.1423 | 11.375 | C11 H20 N2 O4 | RP |
| 1-(2-Furylmethyl)-5-oxopyrrolidine-3-carboxylic acid | 0.582 | 0.015 | 8 | 209.06878 | 3.095 | C10 H11 N O4 | H |
| Hexanoylglycine.1 | 0.578 | 0.043 | 5 | 173.1052 | 10.339 | C8 H15 N O3 | RP |
| (4E)-2-Oxohexenoicacid | 0.571 | 0.001 | 6 | 128.04729 | 9.107 | C6 H8 O3 | RP |
| Ethopabate.1 | 0.566 | 0.001 | 4 | 237.1001 | 11.232 | C12 H15 N O4 | RP |
| Alliin | 0.557 | 0.002 | 7 | 177.04598 | 1.311 | C6 H11 N O3 S | RP |
| O-sebacoylcarnitine | 0.555 | 0.002 | 4 | 345.21517 | 11.966 | C17 H31 N O6 | RP |
| 2-Acetamido-N-fl-aspartyl-2-deoxyhexopyranosylamine | 0.553 | 0.002 | 9 | 335.13302 | 1.105 | C12 H21 N3 O8 | RP |
| (5-methyl-3-isoxazolyl)[4-(5-propyl-2-pyrimidinyl)piperazino]methanone | 0.538 | <0.001 | 6 | 315.16822 | 9.047 | C16 H21 N5 O2 | RP |
| 5-Aminonicotinic acid | 0.535 | 0.013 | 8 | 138.04293 | 2.093 | C6 H6 N2 O2 | H |
| NP-011223.1 | 0.534 | <0.001 | 0 | 208.10993 | 16.839 | C10 H18 O3 | RP |
| 3,5-dibenzylpyridine-2,6-diamine | 0.533 | 0.003 | 0 | 289.15256 | 7.049 | C19 H19 N3 | RP |
| NP-016455 | 0.528 | 0.035 | 5 | 242.12667 | 10.55 | C11 H18 N2 O4 | RP |
| Nitrilotriaceticacid.1 | 0.521 | 0.001 | 5 | 191.04301 | 1.125 | C6 H9 N O6 | RP |
| syringol | 0.519 | <0.001 | 4 | 154.063 | 9.002 | C8 H10 O3 | RP |
| 1,7-Dimethyluric acid | 0.517 | 0.035 | 11 | 196.05966 | 8.881 | C7 H8 N4 O3 | RP |
| 4-Aminohippuricacid | 0.514 | 0.01 | 5 | 194.06917 | 7.857 | C9 H10 N2 O3 | RP |
| 4-Aminohippuric acid | 0.513 | 0.011 | 9 | 194.06913 | 3.217 | C9 H10 N2 O3 | H |
| 2-imino-3a-methylperhydro-5lambda~6~-thieno[3,4-d][1,3 ]thiazole-5,5-dione | 0.512 | 0.001 | 5 | 206.01933 | 9.746 | C6 H10 N2 O2 S2 | RP |
| O-(11-carboxyundecanoyl)carnitine | 0.498 | 0.019 | 4 | 373.24647 | 12.937 | C19 H35 N O6 | RP |
| ophthalmic acid | 0.497 | 0.008 | 4 | 289.12742 | 6.402 | C11 H19 N3 O6 | RP |
| (4S)-4-[(6-Carboxyhexanoyl)oxy]-4-(trimethylammonio)butanoate | 0.484 | 0.01 | 4 | 303.16822 | 8.3 | C14 H25 N O6 | RP |
| (5alpha,6beta)-3-Hydroxy-17-methyl-4,5-epoxymorphinan-6-yl beta-L-glucopyranosiduronic acid | 0.477 | 0.035 | 4 | 463.18424 | 14.471 | C23 H29 N O9 | RP |
| 4-Phenylbutyric acid | 0.473 | 0.007 | 10 | 164.08372 | 10.493 | C10 H12 O2 | RP |
| Hydantoin-5-propionate | 0.472 | 0.014 | 5 | 172.04844 | 1.133 | C6 H8 N2 O4 | RP |
| N-Acetyl-L-phenylalanine | 0.466 | 0.007 | 5 | 207.08953 | 11.731 | C11 H13 N O3 | RP |
| Nitrosoguvacoline | 0.465 | 0.003 | 4 | 170.06924 | 1.39 | C7 H10 N2 O3 | RP |
| Phenylisocyanate | 0.455 | 0.019 | 4 | 119.03707 | 7.857 | C7 H5 N O | RP |
| 4,7-Dioxooctanoic acid | 0.452 | 0.006 | 4 | 172.07353 | 1.889 | C8 H12 O4 | H |
| [Similar to: Hippuric acid; <U+0394>Mass: 179.0585 Da] | 0.45 | 0.015 | 2 | 358.11679 | 3.306 | C18 H18 N2 O6 | H |
| (1S_4S)-4-Hydroxy-3-oxocyclohexane-1-carboxylate | 0.449 | 0.011 | 6 | 158.05786 | 2.361 | C7 H10 O4 | H |
| 3_4-Dihydroxyphenylethyleneglycol | 0.446 | 0.008 | 5 | 170.05791 | 9.126 | C8 H10 O4 | RP |
| Phthaldialdehyde | 0.441 | 0.045 | 6 | 134.03675 | 10.764 | C8 H6 O2 | RP |
| 1_3_6_8-Naphthalenetetrol | 0.439 | <0.001 | 6 | 192.04226 | 12.637 | C10 H8 O4 | RP |
| Buspirone | 0.437 | 0.022 | 7 | 385.24653 | 13.986 | C21 H31 N5 O2 | RP |

| | | | | | | | |
|---|---|---|---|---|---|---|---|
| Hippuric acid | 0.435 | 0.028 | 9 | 179.05824 | 3.308 | C9 H9 N O3 | H |
| Indole-3-acetic acid.1 | 0.435 | 0.005 | 10 | 175.06334 | 10.801 | C10 H9 N O2 | RP |
| cis-2-Carboxycyclohexyl-acetic acid | 0.433 | 0.011 | 4 | 186.08918 | 1.716 | C9 H14 O4 | H |
| 3-amino-2-phenyl-2H-pyrazolo[4,3-c]pyridine-4,6-diol | 0.433 | 0.022 | 0 | 242.07663 | 8.918 | C12 H10 N4 O2 | RP |
| N-[(2S)-2-Hydroxypropanoyl]methionine.2 | 0.43 | 0.029 | 4 | 221.07219 | 7.639 | C8 H15 N O4 S | RP |
| Tetraacetylethylenediamine | 0.427 | 0.002 | 4 | 228.11111 | 1.398 | C10 H16 N2 O4 | RP |
| Methylimidazoleacetic acid | 0.426 | 0.033 | 9 | 140.05857 | 1.322 | C6 H8 N2 O2 | RP |
| N(alpha)-Benzyloxycarbonyl-L-leucine | 0.422 | 0.001 | 5 | 265.13141 | 9.17 | C14 H19 N O4 | RP |
| N-(6-methoxypyridin-3-yl) thiophene-2-carboxamide | 0.421 | 0.002 | 0 | 234.05041 | 12.547 | C11 H10 N2 O2 S | RP |
| 4-(2,3-dihydro-1,4-benzodioxin-6-yl)butanoic acid | 0.421 | 0.027 | 0 | 222.08676 | 10.496 | C12 H14 O4 | RP |
| N-[(2S)-2-Hydroxypropanoyl]methionine | 0.421 | 0.012 | 4 | 221.07221 | 5.854 | C8 H15 N O4 S | RP |
| 4-oxododecanedioic acid.1 | 0.421 | <0.001 | 0 | 266.11543 | 14.777 | C12 H20 O5 | RP |
| 2_6-Dihydroxypseudooxynicotine | 0.419 | 0.004 | 5 | 210.10044 | 7.371 | C10 H14 N2 O3 | RP |
| alpha-D-Xylose1-phosphate | 0.418 | 0.006 | 6 | 230.01917 | 13.283 | C5 H11 O8 P | H |
| XLR11 N-(2-fluoropentyl) isomer.3 | 0.417 | 0.033 | 0 | 329.22029 | 11.569 | C21 H28 F N O | RP |
| O-sebacoylcarnitine.2 | 0.417 | 0.006 | 4 | 345.2152 | 9.449 | C17 H31 N O6 | RP |
| 5-hydroxy-4-methoxy-5,6-dihydro-2H-pyran-2-one | 0.414 | 0.024 | 9 | 144.04228 | 5.851 | C6 H8 O4 | RP |
| Pro-Hyp | 0.41 | 0.004 | 4 | 228.111 | 8.418 | C10 H16 N2 O4 | RP |
| N6,N6,N6-Trimethyl-L-lysine | 0.41 | 0.001 | 10 | 188.15251 | 1.076 | C9 H20 N2 O2 | RP |
| N-(4-Methoxybenzyl)glutamine | 0.409 | 0.008 | 4 | 266.12666 | 6.755 | C13 H18 N2 O4 | RP |
| 1-{(3S,5S)-1-Methyl-5-[3-(4-pyridinyl)-1,2,4-oxadiazol-5-yl]-3-pyrrolidinyl}-3-phenylurea | 0.409 | <0.001 | 0 | 364.16694 | 5.301 | C19 H20 N6 O2 | RP |
| (5-Benzyl-3,6-dioxo-2-piperazinyl) acetic acid | 0.408 | 0.047 | 4 | 262.09534 | 10.498 | C13 H14 N2 O4 | RP |
| thymol sulfate | 0.404 | 0.007 | 4 | 230.06127 | 8.032 | C10 H14 O4 S | RP |
| Ubiquinone-1(CoQ1).1 | 0.403 | 0.007 | 5 | 250.12049 | 16.839 | C14 H18 O4 | RP |
| Proacaciberin.1 | 0.402 | 0.007 | 4 | 391.14793 | 12.076 | C16 H25 N O10 | RP |
| N-Acetyl-D-glucosaminate | 0.4 | 0.015 | 5 | 237.08489 | 1.13 | C8 H15 N O7 | RP |
| Ecgonine | 0.399 | 0.043 | 5 | 185.10515 | 2.956 | C9 H15 N O3 | H |
| 3-Methylhistidine | 0.398 | 0.01 | 7 | 169.08513 | 4.167 | C7 H11 N3 O2 | RP |
| Arenediol | 0.397 | 0.012 | 5 | 112.05238 | 9.002 | C6 H8 O2 | RP |
| 2-(6'-methylthio)hexylmalate.1 | 0.394 | 0.008 | 5 | 264.10332 | 11.456 | C11 H20 O5 S | RP |
| NP-020902 | 0.393 | 0.006 | 0 | 224.06606 | 10.356 | C9 H14 O5 | RP |
| ON1225000 | 0.392 | 0.009 | 4 | 172.07357 | 10.938 | C8 H12 O4 | RP |
| 4,7-Dioxooctanoic acid.1 | 0.387 | 0.006 | 4 | 172.07353 | 1.828 | C8 H12 O4 | H |
| Gentian violet | 0.386 | 0.042 | 0 | 371.23086 | 12.012 | C25 H29 N3 | RP |
| 3-Hydroxy-N-[(3S)-2-oxotetrahydro-3-furanyl]decanamide 3 | 0.384 | 0.005 | 4 | 271.17836 | 11.432 | C14 H25 N O4 | RP |
| NP-016344 | 0.381 | 0.002 | 0 | 244.03818 | 8.905 | C11 H10 O5 | RP |
| Succinyladenosine | 0.38 | 0.005 | 4 | 383.10775 | 8.402 | C14 H17 N5 O8 | RP |
| 3-Methylcrotonylglycine.1 | 0.38 | <0.001 | 6 | 157.0739 | 4.887 | C7 H11 N O3 | RP |
| pentahomomethionine | 0.38 | 0.042 | 6 | 219.1293 | 12.787 | C10 H21 N O2 S | RP |
| 1-(3_4-dimethoxylphenyl)ethane-1_2-diol.1 | 0.379 | 0.01 | 6 | 198.08922 | 12.425 | C10 H14 O4 | RP |
| NP-019445 | 0.378 | 0.015 | 9 | 194.05793 | 12.473 | C10 H10 O4 | RP |
| 4-Hydroxyphenylacetic acid | 0.376 | 0.002 | 9 | 152.04735 | 12.554 | C8 H8 O3 | RP |
| XLR11 N-(2-fluoropentyl) isomer.4 | 0.376 | 0.01 | 0 | 329.22028 | 13.856 | C21 H28 F N O | RP |
| 1,4:3,6-Dianhydro-2-{[4-(1,3-benzodioxol-5-yl)-2-pyrimidinyl]amino}-5-O-(benzylcarbamoyl)-2-deoxy-D-glucitol | 0.374 | 0.019 | 0 | 476.166 | 14.632 | C25 H24 N4 O6 | RP |
| Hydantoin-5-propionate | 0.374 | 0.019 | 5 | 172.04839 | 4.017 | C6 H8 N2 O4 | H |
| N2-(D-1-Carboxyethyl)-L-lysine | 0.371 | 0.036 | 5 | 218.12668 | 1.097 | C9 H18 N2 O4 | RP |
| O-Propanoylcarnitine | 0.371 | 0.021 | 5 | 217.13141 | 13.167 | C10 H19 N O4 | RP |
| 5-Hydroxy-DL-tryptophan | 0.371 | 0.021 | 6 | 220.08473 | 10.498 | C11 H12 N2 O3 | RP |
| N-(5-acetamidopentyl)acetamide.1 | 0.369 | 0.028 | 0 | 208.12116 | 8.501 | C9 H18 N2 O2 | RP |
| N1-[5-(tert-Butyl)-1,3-oxathiol-2-yliden]-2,4,5-trimethylaniline | 0.368 | 0.008 | 0 | 275.1369 | 6.616 | C16 H21 N O S | RP |
| 4-oxododecanedioic acid | 0.366 | 0.012 | 0 | 266.11542 | 13.58 | C12 H20 O5 | RP |
| His-His | 0.366 | 0.007 | 4 | 292.12873 | 14.061 | C12 H16 N6 O3 | RP |
| 4-oxododecanedioic acid.2 | 0.365 | 0.008 | 0 | 266.11435 | 13.018 | C12 H20 O5 | RP |
| thymol sulfate.1 | 0.363 | 0.007 | 4 | 230.06137 | 10.858 | C10 H14 O4 S | RP |
| Propyl gallate | 0.363 | 0.008 | 7 | 212.06847 | 1.806 | C10 H12 O5 | H |

| | | | | | | | |
|---|---|---|---|---|---|---|---|
| (5Z)-13-carboxytridec-5-enoylcarnitine | 0.361 | 0.037 | 4 | 399.26211 | 13.043 | C21 H37 N O6 | RP |
| NP-011223 | 0.359 | 0.018 | 0 | 208.10993 | 17.095 | C10 H18 O3 | RP |
| N'-Cyano-N-[2-(trifluoromethyl) phenyl] pyrrolidine-1-carboximidamide | 0.359 | 0.018 | 5 | 282.10793 | 11.786 | C13 H13 F3 N4 | RP |
| 5-Valerolactone | 0.358 | 0.001 | 5 | 100.05244 | 2.387 | C5 H8 O2 | H |
| Trimethylamine N-oxide | 0.357 | 0.029 | 11 | 75.06844 | 1.162 | C3 H9 N O | RP |
| Tetraacetylethylenediamine.1 | 0.357 | 0.022 | 4 | 228.11111 | 1.726 | C10 H16 N2 O4 | RP |
| NP-021299 | 0.357 | 0.008 | 0 | 482.14025 | 13.579 | C25 H24 N4 O4 | RP |
| Pro-Hyp.1 | 0.354 | 0.001 | 4 | 228.11099 | 8.845 | C10 H16 N2 O4 | RP |
| 6-Hydroxy-1-(hydroxymethyl)-5-{2-[2-(hydroxymethyl)-1-pyrrolidinyl]-2-oxoethyl}-1,4a-dimethyldecahydro-2-naphthalenyl phenylcarbamate | 0.353 | 0.042 | 0 | 510.27642 | 15.191 | C27 H40 N2 O6 | RP |
| Isovalerylglutamic acid.1 | 0.352 | 0.015 | 4 | 231.11068 | 6.559 | C10 H17 N O5 | RP |
| Methohexital | 0.351 | 0.001 | 4 | 262.13177 | 10.208 | C14 H18 N2 O3 | RP |
| 3-Methoxy-4-hydroxyphenylethyleneglycol | 0.35 | 0.014 | 6 | 184.07353 | 1.837 | C9 H12 O4 | H |
| metioprim | 0.35 | 0.005 | 4 | 306.11511 | 6.444 | C14 H18 N4 O2 S | RP |
| 3-Hydroxy-N-[(3S)-2-oxotetrahydro-3-furanyl]octanamide | 0.349 | 0.003 | 4 | 243.14704 | 8.437 | C12 H21 N O4 | RP |
| 3-[(3-Hydroxydecanoyl)oxy]-4-(trimethylammonio)butanoate | 0.349 | 0.021 | 4 | 331.23595 | 12.826 | C17 H33 N O5 | RP |
| NP-019374.2 | 0.348 | 0.029 | 0 | 224.10245 | 11.378 | C10 H18 O4 | RP |
| L-Aspartic acid fl-benzyl ester | 0.347 | 0.015 | 8 | 223.08448 | 10.028 | C11 H13 N O4 | RP |
| NP-021018 | 0.346 | 0.012 | 0 | 208.10995 | 13.022 | C12 H18 O4 | RP |
| Phenylacetyl-L-glutamine | 0.344 | 0.006 | 13 | 264.11097 | 9.953 | C13 H16 N2 O4 | RP |
| Valyl-4-hydroxyproline | 0.34 | 0.01 | 4 | 230.12664 | 8.828 | C10 H18 N2 O4 | RP |
| 5-Aminonicotinic acid.1 | 0.34 | 0.01 | 8 | 138.04293 | 3.193 | C6 H6 N2 O2 | H |
| Nicotianamine | 0.339 | 0.002 | 5 | 303.14306 | 8.858 | C12 H21 N3 O6 | RP |
| 1_6_6-Trimethyl-2_7-dioxabicyclo[3.2.2]nonan-3-one | 0.339 | 0.007 | 5 | 184.10997 | 14.441 | C10 H16 O3 | RP |
| XLR11 N-(2-fluoropentyl) isomer | 0.338 | 0.044 | 0 | 329.22028 | 13.005 | C21 H28 F N O | RP |
| Œ≤-D-Ribofuranosylcreatine | 0.336 | 0.022 | 4 | 263.11175 | 1.324 | C9 H17 N3 O6 | RP |
| Isovalerylglutamic acid.2 | 0.336 | 0.002 | 4 | 231.11068 | 11.486 | C10 H17 N O5 | RP |
| OxidizedRenillaluciferin | 0.335 | 0.028 | 5 | 395.16143 | 12.486 | C25 H21 N3 O2 | RP |
| 5-(acetylamino)-2-hydroxybenzoic acid | 0.333 | 0.001 | 10 | 195.05316 | 9.67 | C9 H9 N O4 | RP |
| Procollagen5-(D-galactosyloxy)-L-lysine | 0.331 | 0.027 | 6 | 324.15335 | 0.957 | C12 H24 N2 O8 | RP |
| 1-Phenyl-1H-pyrazolo[3,4-d]pyrimidin-4-amine.1 | 0.33 | 0.018 | 0 | 211.08206 | 8.902 | C11 H9 N5 | RP |
| trans-Urocanic Acid.1 | 0.33 | 0.021 | 12 | 138.04291 | 2.308 | C6 H6 N2 O2 | RP |
| Hydantoin-5-propionate.1 | 0.33 | 0.015 | 5 | 172.04838 | 10.227 | C6 H8 N2 O4 | H |
| Hippuric acid | 0.33 | 0.025 | 10 | 179.05832 | 9.792 | C9 H9 N O3 | RP |
| 4-chloro-N~1~-(4-pyridinyl)-1,2-benzenediamine | 0.329 | 0.004 | 0 | 219.05315 | 11.364 | C11 H10 Cl N3 | RP |
| N-Acetyl-L-carnosine | 0.328 | 0.006 | 9 | 268.11722 | 11.348 | C11 H16 N4 O4 | H |
| N2-Acetyl-L-aminoadipate.3 | 0.327 | 0.025 | 5 | 203.07936 | 6.615 | C8 H13 N O5 | RP |
| 1,5-Isoquinolinediol | 0.324 | 0.019 | 9 | 161.04771 | 10.398 | C9 H7 N O2 | RP |
| 6-(alpha-D-Glucosaminyl)-1D-myo-inositol | 0.323 | 0.024 | 5 | 341.13236 | 7.647 | C12 H23 N O10 | H |
| 2-Carboxy-2_3-dihydro-5_6-dihydroxyindole | 0.322 | 0.045 | 6 | 195.05317 | 7.38 | C9 H9 N O4 | RP |
| 4-Oxocyclohexanecarboxylate.1 | 0.321 | 0.048 | 5 | 142.06301 | 7.356 | C7 H10 O3 | RP |
| ionene | 0.321 | 0.034 | 4 | 174.14086 | 10.45 | C13 H18 | RP |
| Pantothenicacid(VitaminB5).2 | 0.319 | 0.002 | 6 | 219.11067 | 1.338 | C9 H17 N O5 | RP |
| 3-Hydroxy-N-[(3S)-2-oxotetrahydro-3-furanyl]decanamide.1 | 0.317 | 0.04 | 4 | 271.17836 | 10.648 | C14 H25 N O4 | RP |
| Genipin | 0.315 | 0.043 | 4 | 226.08415 | 1.604 | C11 H14 O5 | H |
| trans-3-Hydroxycotinineglucuronide | 0.314 | 0.013 | 5 | 368.12202 | 10.8 | C16 H20 N2 O8 | RP |
| formyl-isoglutamine.1 | 0.313 | 0.037 | 5 | 174.06409 | 1.295 | C6 H10 N2 O4 | RP |
| ON1225000.1 | 0.313 | 0.007 | 4 | 172.07358 | 9.001 | C8 H12 O4 | RP |
| 3305 | 0.312 | 0.015 | 5 | 126.06804 | 9.003 | C7 H10 O2 | RP |
| 2-(Hydroxymethyl)-1-methyl-3,4,5-piperidinetriol | 0.312 | 0.004 | 4 | 177.10013 | 10.526 | C7 H15 N O4 | RP |
| Isopentenyladenosine | 0.312 | 0.021 | 5 | 335.15818 | 14.187 | C15 H21 N5 O4 | RP |
| (5-Benzyl-3,6-dioxo-2-piperazinyl) acetic acid | 0.311 | 0.034 | 4 | 262.09541 | 6.349 | C13 H14 N2 O4 | H |
| Guanidinosuccinic acid | 0.311 | 0.009 | 9 | 175.05926 | 12.238 | C5 H9 N3 O4 | H |
| Ageratriol | 0.308 | 0.019 | 0 | 234.16199 | 14.558 | C15 H24 O3 | RP |
| NP-001422.1 | 0.308 | 0.021 | 0 | 192.03982 | 10.106 | C8 H10 O4 | RP |

| | | | | | | | |
|---|---|---|---|---|---|---|---|
| N6-threonylcarbamoyladenosine | 0.307 | 0.021 | 4 | 412.13457 | 9.728 | C15 H20 N6 O8 | RP |
| ala-ser | 0.306 | 0.001 | 4 | 176.07975 | 2.435 | C6 H12 N2 O4 | RP |
| Estrone | 0.305 | 0.007 | 0 | 292.13991 | 12.909 | C18 H22 O2 | RP |
| 8'-Hydroxyabscisate | 0.305 | 0.006 | 5 | 280.13109 | 13.581 | C15 H20 O5 | RP |
| Neuraminicacid | 0.304 | 0.021 | 5 | 267.09549 | 1.109 | C9 H17 N O8 | RP |
| N5-(L-1-Carboxyethyl)-L-ornithine | 0.304 | 0.01 | 5 | 204.11109 | 1.383 | C8 H16 N2 O4 | RP |
| Linamarin.1 | 0.302 | 0.009 | 7 | 247.10557 | 11.325 | C10 H17 N O6 | RP |
| Deoxyadenosinemonophosphate | 0.301 | 0.021 | 5 | 331.06689 | 9.037 | C10 H14 N5 O6 P | RP |
| 1-(3-Chlorophenyl)piperazine (m-CPP).1 | 0.297 | 0.013 | 0 | 196.07357 | 15.514 | C10 H13 Cl N2 | RP |
| 2'-fluoro-N,N-diisopropyl[1,1'-biphenyl]-4-carboxamide | 0.297 | 0.021 | 0 | 299.1733 | 9.475 | C19 H22 F N O | RP |
| 8'-Hydroxyabscisate.1 | 0.296 | 0.019 | 5 | 280.1311 | 14.776 | C15 H20 O5 | RP |
| (2S)-3-(1H-Imidazol-4-yl)-2-({[(3S,4S,5R)-2,3,4-trihydroxy-5-(hydroxymethyl)tetrahydro-2-furanyl]methyl}amino)propanoic acid (non-preferred name) | 0.295 | 0.004 | 4 | 317.12253 | 1.396 | C12 H19 N3 O7 | RP |
| NP-016564 | 0.295 | 0.046 | 0 | 290.11306 | 14.982 | C16 H20 O6 | RP |
| Hexanoylglycine.3 | 0.294 | 0.012 | 5 | 173.10527 | 1.393 | C8 H15 N O3 | RP |
| N-Butyryl-L-homoserinelactone.1 | 0.293 | 0.037 | 5 | 171.08957 | 6.56 | C8 H13 N O3 | RP |
| 5-Hydroxypiroxicam | 0.293 | 0.021 | 0 | 347.06176 | 8.685 | C15 H13 N3 O5 S | RP |
| Zalcitabine.1 | 0.293 | 0.013 | 4 | 211.09579 | 1.399 | C9 H13 N3 O3 | RP |
| N-Tigloylglycine | 0.292 | 0.016 | 7 | 157.07387 | 3.47 | C7 H11 N O3 | H |
| (2R,3S,4S,5R,6R)-2-(hydroxymethyl)-6-(2-phenylethoxy)oxane-3,4,5-triol | 0.291 | 0.01 | 0 | 306.108 | 14.776 | C14 H20 O6 | RP |
| JWH-175.1 | 0.291 | 0.014 | 0 | 327.20465 | 11.992 | C24 H25 N | RP |
| 4-Acetamidobutanoic acid | 0.29 | 0.009 | 10 | 145.07387 | 2.418 | C6 H11 N O3 | H |
| 3-Methylcrotonylglycine | 0.289 | 0.028 | 9 | 157.07389 | 8.794 | C7 H11 N O3 | RP |
| Hexanoylcarnitine.1 | 0.287 | 0.012 | 9 | 259.17837 | 10.752 | C13 H25 N O4 | RP |
| (2S)-2-{[1-(R)-Carboxyethyl]amino}pentanoate.2 | 0.287 | 0.01 | 5 | 189.10011 | 9.128 | C8 H15 N O4 | RP |
| Basic Red 9.1 | 0.282 | 0.033 | 0 | 287.13693 | 7.316 | C19 H17 N3 | RP |
| 2-Oxoglutaramate | 0.282 | 0.038 | 8 | 145.03752 | 1.688 | C5 H7 N O4 | RP |
| 2_6-Diaminoheptanedioicacid | 0.281 | 0.023 | 6 | 190.09537 | 1.308 | C7 H14 N2 O4 | RP |
| L-Glutamic acid | 0.277 | 0.008 | 11 | 147.05318 | 1.164 | C5 H9 N O4 | RP |
| 3-Hydroxy-N-[(3S)-2-oxotetra hydro-3-furanyl]decanamide | 0.276 | 0.043 | 4 | 271.17836 | 10.675 | C14 H25 N O4 | RP |
| Glycylprolylhydroxyproline | 0.276 | 0.007 | 4 | 285.13247 | 8.809 | C12 H19 N3 O5 | RP |
| 2-methoxyacetaminophen sulfate | 0.275 | 0.026 | 4 | 261.0307 | 4.73 | C9 H11 N O6 S | RP |
| Azelaicacid.2 | 0.274 | 0.022 | 5 | 188.10486 | 10.414 | C9 H16 O4 | RP |
| 6-Acetamido-3-aminohexanoate | 0.271 | 0.021 | 6 | 188.11609 | 11.136 | C8 H16 N2 O3 | H |
| Eugenol;4-Allylguaiacol | 0.268 | 0.021 | 6 | 164.08373 | 16.893 | C10 H12 O2 | RP |
| (1-Ribosylimidazole)-4-acetate | 0.266 | 0.022 | 5 | 258.08531 | 1.396 | C10 H14 N2 O6 | RP |
| Proline.1 | 0.266 | 0.021 | 11 | 115.06328 | 1.33 | C5 H9 N O2 | RP |
| fl-Cortolone | 0.265 | 0.044 | 0 | 348.23016 | 16.502 | C21 H34 O5 | RP |
| 3-indole carboxylic acid glucuronide | 0.263 | 0.031 | 4 | 337.08014 | 8.152 | C15 H15 N O8 | H |
| [Similar to: N-Acetylneuraminic acid; <U+0394>Mass: -36.0211 Da] | 0.262 | 0.019 | 2 | 273.08492 | 1.325 | C11 H15 N O7 | RP |
| NP-022068.1 | 0.262 | 0.021 | 0 | 210.05041 | 8.426 | C8 H12 O5 | RP |
| EUROPINE.1 | 0.262 | 0.021 | 4 | 329.18394 | 10.141 | C16 H27 N O6 | RP |
| Nitrilotriaceticacid | 0.26 | 0.043 | 5 | 191.04297 | 3.503 | C6 H9 N O6 | RP |
| S-3-oxodecanoyl cysteamine | 0.26 | 0.035 | 4 | 245.14492 | 14.183 | C12 H23 N O2 S | RP |
| NP-016455.1 | 0.26 | 0.019 | 5 | 242.12664 | 10.988 | C11 H18 N2 O4 | RP |
| 12,13-Dimethyl-5,14-dioxabicyclo[9.2.1]tetradeca-1(13),11-dien-4-one.1 | 0.259 | 0.011 | 4 | 236.14123 | 16.844 | C14 H20 O3 | RP |
| 7-mercaptoheptanoylthreonine | 0.258 | 0.033 | 5 | 263.11916 | 13.343 | C11 H21 N O4 S | RP |
| 2-amino-2,3,7-trideoxy-D-lyxo-hept-6-ulosonic acid | 0.257 | 0.048 | 6 | 191.0794 | 8.001 | C7 H13 N O5 | RP |
| 1-methyl-1H-benzimidazole-2-sulfonic acid | 0.256 | 0.038 | 0 | 212.02967 | 4.126 | C8 H8 N2 O3 S | RP |
| 1-Nitrosopiperidine | 0.256 | 0.005 | 4 | 114.07924 | 1.181 | C5 H10 N2 O | RP |
| (8Z,11Z,14Z,17Z,20Z,23Z)-hexacosahexaenoic acid | 0.254 | 0.015 | 4 | 384.3029 | 18.444 | C26 H40 O2 | RP |
| N-Acetyl-L-arginine dihydrate | 0.254 | 0.011 | 12 | 216.12222 | 10.826 | C8 H16 N4 O3 | H |
| O-beta-D-Glucopyranosyl-cis-zeatin | 0.252 | 0.017 | 5 | 381.16364 | 11.966 | C16 H23 N5 O6 | RP |

| | | | | | | | |
|---|---|---|---|---|---|---|---|
| Creatinine | 0.252 | 0.012 | 13 | 113.05887 | 1.203 | C4 H7 N3 O | RP |
| Dopamineglucuronide | 0.251 | 0.045 | 5 | 329.11116 | 10.735 | C14 H19 N O8 | RP |
| D-2-Amino-hexano-6-lactam | 0.245 | 0.012 | 5 | 128.095 | 1.398 | C6 H12 N2 O | RP |
| 4-hydroxy-3-(1-hydroxyiminoethyl)-1,6-diphenyl-1,2-dihydropyridin-2-one | 0.244 | 0.031 | 0 | 320.11093 | 10.277 | C19 H16 N2 O3 | H |
| Guanidinosuccinic acid | 0.243 | 0.021 | 9 | 175.05932 | 1.235 | C5 H9 N3 O4 | RP |
| N-{5-[(dimethylamino)sulfonyl]-2-methylphenyl}cyclohexane carboxamide | 0.242 | 0.013 | 0 | 324.15351 | 15.738 | C16 H24 N2 O3 S | H |
| Limonene-1_2-diol | 0.241 | 0.034 | 5 | 170.13069 | 13.315 | C10 H18 O2 | RP |
| (1-Ribosylimidazole)-4-acetate.2 | 0.241 | 0.01 | 5 | 258.08524 | 5.166 | C10 H14 N2 O6 | RP |
| cis-2-Carboxycyclohexyl-acetic acid.1 | 0.241 | 0.031 | 4 | 186.08919 | 1.4 | C9 H14 O4 | H |
| N6-Acetyl-L-lysine | 0.239 | 0.007 | 11 | 188.11618 | 1.392 | C8 H16 N2 O3 | RP |
| 5-L-Glutamyl-taurine | 0.238 | 0.047 | 5 | 254.05733 | 13.325 | C7 H14 N2 O6 S | H |
| D-Arginine | 0.237 | 0.021 | 6 | 174.11173 | 1.079 | C6 H14 N4 O2 | RP |
| N-Acetyl-1-aspartylglutamic acid | 0.237 | 0.008 | 9 | 304.09067 | 4.592 | C11 H16 N2 O8 | RP |
| Kynurenic acid | 0.234 | 0.033 | 10 | 189.0426 | 9.917 | C10 H7 N O3 | RP |
| 2-imino-3a-methylperhydro-5lambda~6~-thieno[3,4-d][1,3] thiazole-5,5-dione.1 | 0.232 | 0.04 | 5 | 206.01915 | 10.161 | C6 H10 N2 O2 S2 | RP |
| 2_3-Dimethylmaleate | 0.23 | 0.037 | 6 | 144.04224 | 2.09 | C6 H8 O4 | H |
| N-Acetylputrescine | 0.23 | 0.029 | 0 | 89.08411 | 1.394 | C6 H14 N2 O | RP |
| 6-(3Z)-3-Hexen-1-yltetrahydro-2H-Pyran-2-one | 0.23 | 0.015 | 4 | 182.13069 | 13.604 | C11 H18 O2 | RP |
| carglumic acid | 0.229 | 0.037 | 4 | 190.05898 | 1.172 | C6 H10 N2 O5 | RP |
| L(+)-Citrulline;2-Amino-5-uredovalerate.1 | 0.229 | 0.026 | 6 | 175.09577 | 1.396 | C6 H13 N3 O3 | RP |
| 2271703 | 0.228 | 0.021 | 4 | 231.11069 | 11.281 | C10 H17 N O5 | RP |
| N-Feruloylglycine.3 | 0.228 | 0.043 | 5 | 251.07939 | 10.13 | C12 H13 N O5 | RP |
| (+/-)-6-Hydroxy-3-oxo-alpha-ionone | 0.225 | 0.031 | 5 | 222.12558 | 17.094 | C13 H18 O3 | RP |
| N-[(2S)-2-Hydroxypropanoyl]-L-tyrosine | 0.223 | 0.027 | 4 | 253.09501 | 9.101 | C12 H15 N O5 | RP |
| 3-Morpholino-4-tetrahydro-1H-pyrrol-1-ylcyclobut-3-ene-1,2-dione | 0.215 | 0.029 | 0 | 236.1137 | 8.729 | C12 H16 N2 O3 | RP |
| beta-Aspartylaspartic acid | 0.213 | 0.049 | 4 | 248.06446 | 1.249 | C8 H12 N2 O7 | RP |
| MFCD02728197 | 0.212 | 0.04 | 4 | 280.14236 | 8.968 | C14 H20 N2 O4 | RP |
| Boc-Asn-ONp | 0.21 | 0.019 | 4 | 353.12241 | 7.367 | C15 H19 N3 O7 | RP |
| Indole-3-acetaldoxime | 0.207 | 0.034 | 5 | 174.07932 | 5.56 | C10 H10 N2 O | RP |
| 4,7-dimethylpyrazolo[5,1-c][1,2,4]triazine-3-carbonitrile | 0.199 | 0.041 | 0 | 173.06879 | 2.59 | C8 H7 N5 | H |
| Ubiquinone-2(CoQ2) | 0.199 | 0.019 | 5 | 318.18316 | 17.811 | C19 H26 O4 | RP |
| NP-020014.1 | 0.198 | 0.04 | 0 | 236.17762 | 16.985 | C15 H26 O3 | RP |
| 7a-Hydroxytestosterone.1 | 0.196 | 0.01 | 10 | 304.20386 | 15.775 | C19 H28 O3 | RP |
| Cantharidin.1 | 0.183 | 0.044 | 9 | 196.07355 | 13.487 | C10 H12 O4 | RP |
| Deoxyadenosinemonophosphate.1 | 0.178 | 0.033 | 5 | 331.06695 | 7.737 | C10 H14 N5 O6 P | RP |
| [Similar to: Uric acid; <U+0394>Mass: 192.0271 Da] | -0.18 | 0.021 | 2 | 360.05546 | 2.799 | C5 H13 N8 O9 P | RP |
| Betaine | -0.29 | 0.001 | 14 | 117.07892 | 1.187 | C5 H11 N O2 | RP |
| 20 fl-Dihydrocortisol.1 | -0.29 | 0.021 | 8 | 364.22507 | 15.342 | C21 H32 O5 | RP |
| Stachydrine | -0.3 | 0.028 | 6 | 143.09463 | 7.831 | C7 H13 N O2 | RP |
| Choline | -0.31 | 0.001 | 7 | 103.09968 | 1.099 | C5 H13 N O | RP |
| Capryloylglycine | -0.32 | 0.035 | 5 | 201.13646 | 15.199 | C10 H19 N O3 | RP |
| Capryloylglycine | -0.33 | 0.019 | 6 | 201.13648 | 1.929 | C10 H19 N O3 | H |
| Pyridoxamine;2-Methyl-4-aminomethyl-5-hydroxymethyl-3-Pyridinol | -0.34 | 0.027 | 6 | 168.08986 | 7.406 | C8 H12 N2 O2 | RP |
| fosfocreatinine | -0.35 | 0.007 | 4 | 193.02537 | 1.071 | C4 H8 N3 O4 P | RP |
| Myristamide | -0.36 | 0.041 | 4 | 227.22491 | 20.297 | C14 H29 N O | RP |
| 3-(propan-2-yl)-octahydropyrrolo [1,2-a]pyrazine-1,4-dione | -0.41 | 0.009 | 6 | 196.12117 | 10.008 | C10 H16 N2 O2 | RP |
| Dihydrothymine | -0.47 | 0.013 | 9 | 128.05854 | 1.256 | C5 H8 N2 O2 | RP |
| hypaphorine | -0.47 | 0.007 | 4 | 246.1368 | 8.79 | C14 H18 N2 O2 | RP |
| PEG n6 | -0.5 | 0.045 | 7 | 282.16792 | 9.715 | C12 H26 O7 | RP |
| PEG n7 | -0.66 | 0.023 | 7 | 326.19414 | 10.338 | C14 H30 O8 | RP |

| | | | | | | | |
|---|---|---|---|---|---|---|---|
| (6R,7S)-6,7-Dihydroxy-8-methyl-8-azabicyclo[3.2.1]oct-3-yl (2E)-2-methyl-2-butenoate | -0.9 | 0.024 | 4 | 255.14706 | 12.842 | C13 H21 N O4 | RP |
| 2-(4-Isobutylphenyl)acrylic acid | -0.91 | 0.045 | 4 | 204.11501 | 13.44 | C13 H16 O2 | RP |
| 5,6-Dihydroxy-2-naphthalene sulfonic acid | -1.16 | <0.001 | 4 | 240.00927 | 1.15 | C10 H8 O5 S | H |

Supplemental Table 2 In-depth Descriptions of Features from Table 2

| Metabolite | Relevance |
|---|---|
| Taurine | The most abundant free amino acid in human cells; synthesized from other sulfonic amino acids such as methionine and cysteine, must also be provided by diet. It participates in bile acid formation, exerts antioxidant and anti-inflammatory actions as well as antiarrhythmic, ionotropic and chronotropic ones, is a central nervous system neuro-modulator, and is involved in retinal development and function. [53] |
| | There is an inverse association between taurine levels in urine and risk of mortality from coronary heart disease and stroke in adults aged 48-56. High urinary taurine/creatinine ratios were also inversely associated with cardiovascular disease risk factors, including BMI, BP, TC, obesity, hypertension and hypercholesterolemia. Taurine deficiency was associated with higher risk of hypertension. [54] |
| | Taurine is produced by the liver in response to exposure to toxins--increased concentration in plasma and urine can be used as a biomarker for paracetamol poisoning, and can also result from surgical trauma, X-radiation, muscle necrosis, or carbon tetrachloride-induced liver damage. [55] |
| 1,7-Dimethyluric acid | conversion between caffeine metabolite 1,7-dimethylxanthine to 1,7-dimethyluric acid is being used to represent CYP2A6 activity which may represent a marker for susceptibility to dietary carcinogens; CYP2A6 bioactivates nitrosamines. [56] |
| 4-Phenylbutyric acid | Benzene butyric acid may be potential biomarkers for early inflammation-stage pulmonary fibrosis. [57] |
| | Used clinically to treat urea cycle disorders, also prevents misfolded protein aggregation and alleviates endoplasmic reticulum stress. [58] |
| Indole-3-acetic acid | Indole-3-acetic acid is a naturally abundant auxin, a regulator of plant growth. It is found in human urine, plasma and the nervous system due to heavy consumption of vegetables, production by intestinal bacteria, or synthesis from tryptophan in tissues. [59] |
| | Indole-3-acetic acid concentration in urine correlates with liver damage and non-alcoholic steatohepatitis (NASH). It can also be used as a biomarker to differentiate between NASH and non-alcoholic fatty liver disease (NAFLD). [60] |
| | Testing for Indole-3-acetic acid in spot urine can be reliably used to diagnose acute appendicitis. [61] |
| N6,N6,N6-Trimethyl-L-lysine | Non-protein amino acid, starting point for carnitine biosynthesis in mammals, essential for fatty acid transport and use in mitochondria. Produced by hydrolysis of proteins containing N6,N6,N6-Trimethyl-L-lysine as a post-translational modification of some lysine residues.[62] |
| | High concentration of N6, N6, N6-trimethyl-L-lysine in urine is a potential high-risk biomarker for bladder cancer. [63] |
| | N6,N6,N6-Trimethyl-L-lysine (TML) levels are independently associated with risk of incident (3-year) major adverse cardiovascular events and risk of incident (5-year) mortality. TML levels can be used as a nutrient precursor for gut microbiota–dependent generation of trimethylamine (TMA) and trimethylamine N-oxide (TMAO). [64] |
| Trimethylamine N-oxide | Increasing interest in understanding importance of TMAO relationships and dynamics with NOC formation, carcinogenic potential via DNA-methylation and direct mutagenic properties. [65] Noted uremic toxin[25] |
| Phenylacetyl-L-glutamine | Phenylacetylglutamine is a colonic microbial metabolite from amino acid fermentation; it results from glutamine conjugation of phenylacetic acid. Higher concentrations of phenylacetyl-l-glutamine is found in serum of patients with advanced chronic kidney disease, and is a risk factor for cardiovascular disease and mortality. [66] |

| | |
|---|---|
| | Phenylacetylglutamine is a potential biomarker for ischemic stroke and is associated with unfavorable short-term outcomes. [67] |
| | 5-(acetylamino)-2-hydroxybenzoic acid |
| 5-(acetylamino)-2-hydroxybenzoic acid | Metabolite of 5-Aminosalicylic acid (mesalazine, 5-ASA, drug used to treat inflammatory bowel diseases); therepeutically inert, major metabolite present in blood, binds to proteins. [68] |
| trans-Urocanic Acid.1 | following exposure to UVR, the naturally occurring trans-UCA is converted to cis-UCA, both of which have shown contributing to both health and disease; UV-induced cis-UCA is suggested to promote skin cancer by reducing cell-mediated immunity. [69] |
| Hippuric acid | Useful as a marker for bacterial overgrowth in the intestines, linked to polyphenol-rich diet; differences in green tea and black tea phenol production illustrate large interindividual variability between phenolic metabolite production and hippuric acid presence highlighting importance of individual microflora. [70] |
| 4-Acetamidobutanoic acid | Predominant inhibitory neurotransmitter in central nervous system in mammals, also produced in plants to mitigate stress. It also plays a role in the induction of hypotension, and has tranquilizer and diuretic effects. Produced by decarboxylation of glutamic acid. [71] |
| | Used as a general biomarker for alcoholic liver injury, sepsis, malnutrition, multiple organ failure; linked to tuberculosis and pediatric metabolic diseases. May also be used as a biomarker for osteoporosis. [72] |
| L-Glutamic acid | discusses large set of urinary metabolites including glutamic acid, glutamine and derivates that were observed to respond significantly after neoplasm. [73] |
| | highlights glutamic acid's anticancer properties; cellular life and proliferation relies greatly on glutamine metabolic pathway and glutamic acid; endogenous levels of glutamic acid suggested to ameliorate cancerous side effects; in cancer patients, the deficiency of glutamate, glutamine, and other associated metabolites was suggested contribute to depletion of malignant cells. [74] |
| Proline | a precursor for hydroxyproline and used to produce collagen - proline and hydroxyproline urinary excretion can then be used as an index of collagen catabolism; collagen may promote cancer cell growth and migration during tumor-specific extracellular matrix remodeling. [75] |
| N-Acetyl-L-arginine dihydrate | known human metabolite; N-Acetylated-L-arginine (NALA) enhanced properties for arginine as an aggregation suppressor while minimizing disturbance of protein conformational stability. [76] |
| Creatinine | in addition to representing kidney health, serum creatinine levels may prove useful as a prognostic parameter predicting disease survival in vulvar cancer. [77] |
| | renal monitoring for all cancer types is recommended measuring creatinine clearance through 24-hour urine collection. [78] |
| N6-Acetyl-L-lysine | role as human metabolite; lysine acetylation occurs for both histone and non-histone proteins throughout the cell and has great impact on protein functioning; article discusses implications of lysine acetylation in various cellular compartments associated with cancer biology and relevance of lysine acetylation as biomarkers for cancer prognosis. [79] |
| | highlight novel potential cancer drivers for use as biomarkers and propose a new tumorigenesis mechanism tracking misregulation of lysine modifications in cancer-relavent pathways. [80] |
| Kynurenic acid | KYNA is a metabolite of L-tryptophan and is associated with non-recovery from acute renal failure. It is also involved in AhR pathway activation. KYNA may represent an important cancer marker: easily absorbed through digestive tract - can quickly concentrate serum and organs; pro-carcinogenic properties; endogenously produced by normal and cancer cells. [81] |

| | |
|---|---|
| 7a-Hydroxytestosterone | using urine metabolomics to conduct  a biomarker exploration for gestational diabetes mellitus and spontaneous abortion, results show steroid hormone metabolites were significantly regulated and consistently upregulated in pregnant women. [82] |
| Betaine | Methyl derivative of glycine; functions as an organic osmolyte to protect cells under stress, acts as a catabolic source of methyl groups via transmethylation for use in various biochemical pathways. [83] |
| | Betaine in urine can be used as a dietary biomarker, especially of citrus fruit, and is associated with a decrease of inflammation. [84] |
| | Betaine concentration is inversely associated with risk of breast cancer. [85] |
